# Mitochondrial Resilience in Glaucoma: Targeting NAD^+^ Metabolism and Oxidative Stress in Retinal Ganglion Cell Degeneration with Nicotinamide Riboside and Berberine: Preliminary Clinical Evidence

**DOI:** 10.3390/diseases14020056

**Published:** 2026-02-02

**Authors:** Federico Visalli, Francesco Cappellani, Giuseppe Gagliano, Alfonso Spinello, Alessandro Avitabile, Ludovica Cannizzaro, Matteo Capobianco, Caterina Gagliano, Marco Zeppieri

**Affiliations:** 1 Department of Ophthalmology, University of Catania, 95123 Catania, Italy; federico.visalli13@gmail.com (F.V.);; 2 Department of Medicine and Surgery, University of Enna “Kore”, Piazza dell’Università, 94100 Enna, Italy; 3 Mediterranean Foundation “G.B. Morgagni”, 95125 Catania, Italy; 4 Faculty of Medicine, University of Catania, 95123 Catania, Italy; 5 Department of Ophthalmology, University Hospital of Udine, 33100 Udine, Italy; 6 Department of Medicine, Surgery and Health Sciences, University of Trieste, 34127 Trieste, Italy

**Keywords:** glaucoma, nicotinamide riboside, nicotinamide, berberine, NAD^+^ metabolism, mitochondrial dysfunction, retinal ganglion cells, neuroprotection, oxidative stress

## Abstract

Background: Glaucoma is a chronic neurodegenerative disorder characterized by the selective vulnerability of retinal ganglion cells (RGCs), in which mitochondrial dysfunction, redox imbalance, and impaired bioenergetic signaling play central pathogenetic roles. Mitochondrial homeostasis in RGCs critically depends on maintaining intracellular NAD^+^ pools, which support oxidative phosphorylation, sirtuin-mediated deacetylation, and antioxidant gene expression. Nicotinamide riboside (NR), a potent NAD^+^ precursor, and berberine (BBR), an AMPK activator derived from Berberis aristata, have recently emerged as synergistic modulators of mitochondrial metabolism and oxidative stress resistance. Methods: This study retrospectively assessed clinical outcomes associated with combined nutraceutical supplementation of nicotinamide riboside (NR) and berberine (BBR) in patients with primary open-angle glaucoma undergoing stable topical hypotensive therapy. We have included a narrative review in the current literature regarding NAD^+^ biology, AMPK–sirtuin signaling, and oxidative stress responses in retinal ganglion cell (RGC) degeneration. Due to the absence of comparator groups receiving only NR or only berberine in this retrospective cohort, the combined supplementation has been regarded as a biologically complementary strategy, and the potential for synergistic efficacy remains a subject for further investigation. Results: Translationally, a retrospective clinical cohort receiving combined NR and BBR supplementation showed functional stabilization of the visual field and structural preservation of the retinal nerve fiber layer over a six-month follow-up, in line with the proposed mitochondrial protective mechanisms. Conclusions: The clinical trends identified in this retrospective cohort have substantiated the translational significance of NR + BBR supplementation as a potential adjunctive approach in glaucoma management. NAD^+^ repletion and engagement of the AMPK–SIRT–NRF2 pathway may enhance mitochondrial resilience in RGCs. Collectively, these findings offer initial clinical evidence advocating for additional controlled studies on NR + berberine supplementation, while mechanistic interpretations have been derived from the existing literature and are hypothesis-generating.

## 1. Introduction

Glaucoma is a progressive optic neuropathy and one of the leading causes of irreversible blindness worldwide [1]. One of the pathophysiological causes of glaucoma is retinal ganglion cell (RGC) degeneration [2]. Damage to the RGCs and their axons causes characteristic defects that can be visualized in the visual field and represent the characteristic damage in glaucoma [3]. In many patients, loss of RGCs and deterioration of the visual field can be observed despite intraocular pressure (IOP) within the “normal ranges” range.

Despite this evidence, high intraocular pressure is currently the main modifiable risk factor in patients with glaucoma. Although elevated intraocular pressure (IOP) is the main modifiable risk factor for glaucoma, many patients continue to experience RGC loss and vision deterioration despite well-controlled IOP [4]. These clinical findings indicate that other factors, in addition to the mechanical stress resulting from elevated IOP, contribute to glaucomatous neurodegeneration. In this way, it is possible to assume a view of glaucoma as a neurodegenerative disease of the visual system rather than a disorder purely secondary to ocular hypertension [2]. In support of a neurodegenerative role, it is evident that glaucomatous damage extends along the visual pathway in the brain in a manner similar to that observed in other neurodegenerative conditions; moreover, apoptosis of RGCs is an early and fundamental event in the progression of glaucomatous disease [5].

Among the main pathogenetic mechanisms underlying damage and death by RGC are chronic oxidative stress and mitochondrial dysfunction [6]. In glaucoma, ischemia-reperfusion damage to the optic nerve head (ONH) due to impaired ocular blood flow or transient spikes in IOP leads to the overproduction of reactive oxygen species (ROS) and a state of oxidative stress that directly damages the RGCs [7]. The optic nerve head and retina have a high metabolic state, and RGC axons have a huge energy demand; as a consequence, any alteration in mitochondrial function can compromise the survival of RGCs [8]. In recent years, mitochondrial dysfunction has been increasingly recognized as an important factor contributing significantly to the genesis of glaucomatous neuropathy [6]. This is supported by the accumulation of abnormal mitochondria in the ONH and by the increased susceptibility of aging RGCs to mitochondrial dysfunction with reduced mitochondrial reserve [9].

High oxidative stress and insufficient mitochondrial energy production form a vicious cycle that impairs axonal transport and promotes RGC apoptosis and progressive neurodegeneration in glaucoma [9]. This evidence on the pathophysiology of glaucoma has put oxidative damage and mitochondrial bioenergetic failure as primary targets for neuroprotective intervention in glaucoma patients [10]. Given the multifactorial nature of glaucoma pathogenesis, there has been increasing interest in neuroprotective strategies that go beyond IOP lowering to directly preserve RGCs and their function. To date, no definitive neuroprotective therapy has become standard in clinical practice. Pharmacological agents such as brimonidine have demonstrated non-negligible protective effects on RGCs in animal models of glaucoma regardless of IOP reduction [11]. In particular, glaucomatous eyes treated with brimonidine supplementation had increased RGC survival and reduced axonal degeneration even when IOP was not elevated [12].

In this context, supplementation with nicotinamide adenine dinucleotide (NAD^+^) has attracted considerable attention as an innovative approach to support RGC metabolism [13]. Nicotinamide riboside (NR) is a precursor of NAD and is emerging as a potent mitochondrial biomodulator with neuroprotective effects in patients with glaucoma. Adequate supplementation is essential for mitochondrial function, energy production (ATP) and cellular resistance to biomechanical stress. It should also be noted that NAD levels tend to decrease with increasing age. All this leads to chronic oxidative damage [14]. In experimental models, strategies that increase NAD levels have created significant benefits. In particular, dietary nicotinamide supplementation has been shown to strengthen mitochondrial robustness in RGCs and delay glaucoma-related neurodegeneration [15].

Williams et al. have shown that supplementation with high doses of nicotinamide in aged mice ensured consistently high NAD levels, which protected RGCs from axonal damage and apoptosis [16]. In this way, it is possible to prevent the progression of glaucoma in mice. As a result, an increasing number of studies are investigating the role of NAD in humans. A recent study found that patients with primary open-angle glaucoma have significantly lower plasma concentrations of nicotinamide than age-matched controls [17]. This suggests that a systemic deficiency of NAD may be linked to susceptibility to glaucoma. In addition, a randomized, placebo-controlled trial reported that oral nicotinamide (3 g/day) produced a measurable improvement in IOP in patients with glaucoma on standard therapy [18]. In particular, electrophysiological assessments (ERG patterns) indicated an improvement in RGC function after several weeks of high-dose nicotinamide supplementation, providing tangible clinical evidence of the neuroprotective effect of nicotinamide [18].

Nicotinamide, and by extension nicotinamide riboside, a precursor of NAD with greater bioavailability, is an ideal candidate for neuroprotection in patients with glaucoma. Nicotinamide targets mitochondrial energy failure that contributes to RGC vulnerability. Finally, its favorable safety profile and the benefits observed in other diseases, such as Alzheimer’s and Parkinson’s disease models, further strengthen the rationale for its use in glaucomatous optic neuropathy [19,20].

In parallel to nicotinamide, another nutraceutical agent—berberine—is attracting increasing interest as a therapy targeting mitochondrial dysfunction with antioxidant and neuroprotective properties [21,22]. Berberine is a naturally occurring isoquinoline alkaloid (extracted from plants such as Berberis). Berberine exerts a wide range of beneficial effects on metabolism and neuronal health [23]. Crucially, berberine can cross the blood–brain barrier to exert its effects directly within the central nervous system [24]. In particular, berberine exhibits significant neuroprotective efficacy in several preclinical models of neural damage, including ischemia-induced brain damage, ROS-induced neurotoxicity, and chronic neurodegenerative diseases [25].

The mechanisms underlying the neuroprotective action of berberine are many: on the one hand, it works as a powerful antioxidant, reducing ROS-induced damage, and has anti-inflammatory activity, dampening microglial activation and the release of pro-inflammatory cytokines; on the other hand, it is able to inhibit pro-apoptotic pathways by promoting cell survival signaling [25]. In studies of neuronal cell cultures, berberine has been shown to maintain mitochondrial membrane potential, reduce excitotoxic glutamate release, and modulate key stress-response kinases, thereby preserving neuronal viability across various pathological conditions [26]. It should also be emphasized that the protective effects of berberine extend directly to retinal neurons. In an in vivo study of retinal degeneration, systemic treatment with berberine significantly increased RGC survival, reduced RGC apoptosis, and improved visual function, as measured by electrophysiological examinations [27]. This finding, albeit in a model of diabetic retinopathy, underscores berberine’s ability to support retinal ganglion cell health and suggests a potential application to glaucoma, in which RGC degeneration is the primary feature. To date, however, direct clinical evidence for berberine in glaucomatous disease is limited.

Most human research on berberine has focused on metabolic and neurodegenerative disorders such as diabetes, Alzheimer’s, and Parkinson’s disease [21,28]. Its role in glaucoma remains largely theoretical; however, it is supported by biological mechanisms and preclinical data, but is not yet confirmed in patient studies. Nevertheless, the pharmacological profile of berberine makes it an attractive candidate for mitochondrial biomodulation therapy in glaucoma neuroprotection. Berberine’s favorable safety profile as an oral supplement and its low systemic toxicity further support its consideration for chronic neuroprotective use [29].

Based on the discussion above, the aim of the present study was to evaluate the potential therapeutic effect of combined nicotinamide riboside and berberine supplementation in patients with glaucoma. We performed a retrospective clinical analysis to investigate whether the combined supplementation of nicotinamide riboside and berberine can confer measurable neuroprotective benefits in glaucoma patients. Specifically, the study examined the effects of chronic supplementation with nicotinamide riboside and berberine on indicators of RGC disease, including disease progression. We analyzed visual field stability, thinning of the retinal nerve fiber layer, or electrophysiological function of patients with primary open-angle glaucoma who underwent topical hypotonic therapy. By analyzing clinical data, we aimed to determine whether mitochondrial biomodulation induced by NR and berberine supplementation is associated with protection against neurodegeneration and/or improved retinal function. In this way, preliminary clinical information can be provided on their efficacy as neuroprotective agents. Below, we report our results on the outcomes of NR and berberine supplementation in glaucoma, with the aim of contributing to the evolution of the paradigm of neuroprotective therapy of glaucoma.

This study was not designed to investigate mechanisms at the molecular or experimental level; consequently, no direct analyses of intracellular signaling or retinal cell models were performed. Instead, the present work aims at retrospective clinical observation, which is interpreted within a translational framework informed by previously published mechanistic studies. Consequently, references to intracellular pathways and mitochondrial signaling are proposed as biologically plausible mechanisms aimed at supporting hypothesis generation, rather than representing a mechanistic validation within the analyzed cohort.

### Cellular and Molecular Mechanisms of Mitochondrial Biomodulation

At the molecular level, the neuroprotective effects of nicotinamide riboside and berberine are mediated by a finely orchestrated network of intracellular pathways that regulate mitochondrial bioenergetics, oxidative balance, and neuronal survival signaling in retinal ganglion cells (RGCs) [30]. These mechanisms together enhance mitochondrial resistance and counteract the bioenergetic and oxidative stress that drive glaucomatous neurodegeneration [30].

Nicotinamide riboside (NR) works primarily by restoring intracellular levels of nicotinamide adenine dinucleotide (NAD^+^). NR therefore represents an important support for both mitochondrial respiration and for the activation of NAD^+^-dependent enzymes, in particular SIRT1 and SIRT3 [31]. Within the nucleus, SIRT1 deacetylates and activates PGC-1α (peroxisome proliferator-activated receptor gamma coactivator 1-alpha), which represents an important regulator of mitochondrial biogenesis and oxidative metabolism [31]. This activation promotes the transcription of mitochondrial genes involved in energy production and antioxidant defense. In parallel, mitochondrial SIRT3 deacetylates key enzymes in the electron transport chain and the antioxidant enzyme MnSOD (SOD2) [31]. This results in more efficient electron transport and a reduction in mitochondrial ROS.

Through these converging effects, NR enhances oxidative phosphorylation, stabilizes mitochondrial dynamics, and protects RGCs from apoptosis resulting from reduced energy levels [32]. In addition, elevated NAD^+^ levels are also important to ensure genomic stability by supporting cell signaling pathways implicated in primarily PALP-mediated DNA repair without depleting NAD^+^ cell pools, and enhance FOXO3a-dependent transcription of genes involved in detoxification and stress resistance, further increasing mitochondrial integrity [32].

Berberine (BBR) acts by activating the AMP-activated protein kinase (AMPK) pathway, an important mitochondrial metabolic sensor that responds to low energy levels [33]. Upon activation, AMPK phosphorylates and activates PGC-1α, which promotes mitochondrial biogenesis and improves metabolic efficiency. AMPK also upregulates nicotinamide phosphoribosyltransferase (NAMPT), which is the limiting enzyme in NAD^+^ biosynthesis. This further strengthens the NAD^+^/SIRT1 signaling cycle initiated by NR. At the same time, AMPK can inhibit mTOR activity, which is implicated in autophagy and mitophagy [33]. Such processes are critical for maintaining a healthy mitochondrial population, as they primarily remove damaged or nonfunctional mitochondria [25]. The control of mitochondrial well-being is particularly relevant in conditions of glaucomatous stress. In fact, the accumulation of dysfunctional organelles is a key factor in axonal degeneration. Berberine also exerts potent antioxidant and anti-inflammatory effects through modulation of the NRF2-Keap1 signaling axis. Berberine also activates AMPK by promoting the dissociation of NRF2 from its cytoplasmic inhibitor Keap1, so that NRF2 can translocate within the nucleus. At this level, NRF2 is free to bind to antioxidant response elements (AREs) and to upregulate the transcription of cytoprotective genes such as heme oxygenase-1 (HO-1), NAD(P)H quinone oxidoreductase-1 (NQO1), and glutathione peroxidase (GPx) [34]. In this way, berberine enhances the redox buffering capacity of RGCs and mitigates ROS-mediated damage. In addition, berberine appears to suppress the activation of NF-κB and NLRP3-mediated inflammatory pathways, thereby also reducing microglial activation and the release of pro-inflammatory cytokines in the glaucomatous retina [25].

## 2. Results

**Figure 1:**
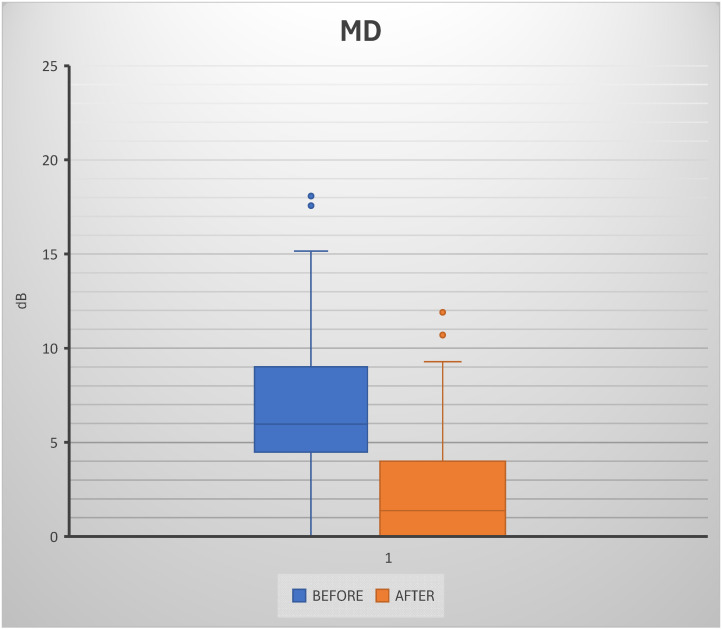
The box illustrates the distribution of mean deviation (MD) values before and after treatment. The data show a reduction in median MD values in the post-treatment group relative to the pre-treatment group, with a narrower interquartile range and lower overall variability. A few outliers were observed in both groups, although they were less pronounced after treatment. Overall, the plot highlights a statistically relevant functional improvement following the intervention.

After six months of consistent supplementation with nicotinamide riboside and berberine, approximately 70% of patients showed stabilization or a slight improvement in mean deviation (MD) of the visual field from baseline. In patients taking combined supplementation, the characteristic progressive decline in MD associated with glaucoma was halted or modestly reversed. Statistical analysis revealed a significant reduction in mean deviation (MD) values to the visual field after 6 months of treatment. The overall mean MD was 7.20 ± 4.54 dB, indicating a substantial functional change in the study population. Comparing pre- and post-treatment conditions, Student’s *t*-test demonstrated a highly significant difference (*p* = 3.13 × 10^−10^), strongly supporting the hypothesis of treatment-related improvement. Narrowing of the interquartile range in the post-treatment group further supports the consistency of the observed effect, with a decrease in variability relative to baseline. Although a limited number of outliers were detected, their influence did not alter the overall statistical significance. These results suggest that the intervention not only improved median MD values but also led to more homogeneous functional outcomes among patients. Overall, the results provide strong evidence of a clinically and statistically relevant benefit associated with treatment (Figure 1).

**Figure 2:**
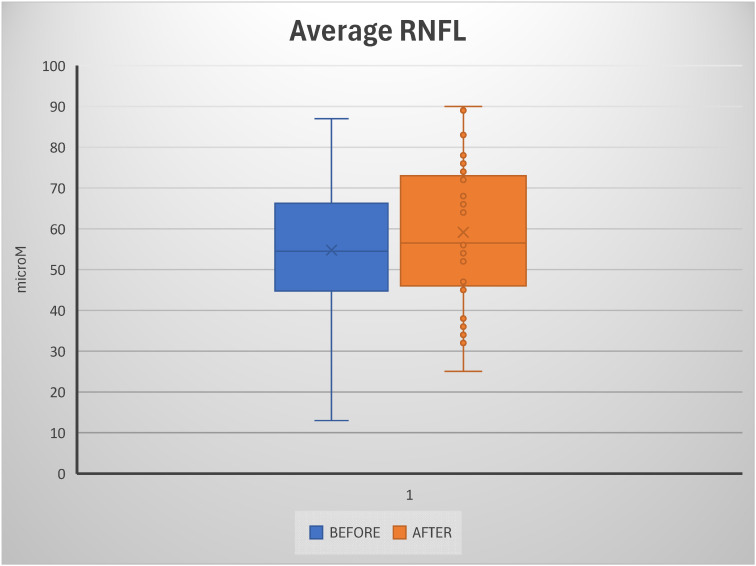
Box plot showing the distribution of average retinal nerve fiber layer (RNFL) thickness before (blue) and after (orange) treatment. The post-treatment group had a slightly higher median thickness than the pre-treatment group, along with a wider interquartile range and several outliers, indicating greater variability across subjects. In contrast, the pre-treatment values appeared more concentrated around the median, with a broader extension toward lower RNFL thickness values. Overall, the figure suggests a trend toward increased average RNFL thickness following treatment, although this is accompanied by greater dispersion in the measurements.

A subset of patients, whom we termed “responders”, also showed a positive trend of increased RNFL thickness on OCT, or at least a halt in RNFL thinning, compared with pre-treatment measurements. Analysis of retinal nerve fiber layer (RNFL) thickness revealed a significant difference between pre- and post-treatment conditions. The mean RNFL thickness increased from 54.78 ± 16.32 μm at baseline to 59.11 ± 16.80 μm after treatment. This difference was statistically significant, as confirmed by the Student’s *t*-test (*p* = 1.89 × 10^−7^). The increase in mean values suggests a measurable effect of the intervention on RNFL preservation or enhancement. Although the standard deviation remained relatively high in both groups, indicating considerable inter-individual variability, the overall distribution showed a consistent upward shift in post-treatment measurements. This was also reflected in the box plot, which showed a higher median and a broader interquartile range after treatment. Despite the statistical significance of this increase, its biological interpretation should be approached with caution, given the well-established test–retest variability of OCT-derived RNFL measurements, particularly in eyes with advanced thinning and increased susceptibility to segmentation artifacts. Outliers were more frequent in the post-treatment group, but their presence did not alter the robustness of the statistical significance. Collectively, these findings support the hypothesis that the treatment exerts a positive impact on RNFL thickness, with potential functional implications (Figure 2).

This tendency of a thicker RNFL, even if thinner than normal, may indicate a slowing of axonal loss and a potential structural benefit to the optic nerve. By contrast, in the remaining approximately 30% of patients, there was no evidence of these benefits. These patients showed a gradual deterioration in visual field or no significant change in RNFL measurements. Our clinical experience suggests that combined supplementation with nicotinamide riboside and berberine mitigates RGC neurodegeneration. Clinically, this is evidenced by stabilization or non-improvement of the visual field MD in most patients and a slight thickening of the RNFL in some patients. Certainly, further controlled studies will be needed to confirm these preliminary findings, thereby determining statistical significance and clarifying which patient subgroups are most likely to benefit from this supplementation intervention. Table 1 summarizes retinal nerve fiber layer (RNFL) thickness and mean deviation (MD) before and after treatment.

## 3. Discussion

Given the complex, multifactorial nature of glaucoma pathogenesis, there has been a growing shift toward neuroprotective strategies that extend beyond lowering intraocular pressure (IOP). Increasingly, the focus is on preserving the health and function of retinal ganglion cells themselves. This evolving perspective is grounded in a body of experimental and translational research suggesting that factors such as metabolic insufficiency, oxidative stress, mitochondrial dysfunction, and chronic neuroinflammation may drive disease progression, even when IOP is well controlled [34].

Against this backdrop, interest in neuroprotective interventions and targeted drug delivery systems for retinal neurons has grown substantially. In particular, therapies that address mitochondrial health are gaining traction as promising adjunctive options in the effort to slow or halt glaucomatous neurodegeneration [35].

Given the multifactorial nature of glaucoma and the ongoing loss of retinal ganglion cells (RGCs) despite seemingly adequate intraocular pressure (IOP) control, neuroprotective strategies that integrate metabolic support with continuous local pharmacological administration have been increasingly investigated as adjuncts to conventional hypotensive treatment. Luo and colleagues created an injectable, biodegradable thermoresponsive hydrogel derived from benzoic acid-modified chitosan-g-poly (N-isopropylacrylamide), intended for prolonged intracameral drug release while concurrently demonstrating intrinsic antioxidant properties via methoxylation-dependent redox modulation [36]. This platform has facilitated the co-delivery of pilocarpine and the histone deacetylase inhibitor RGFP966, leading to significant reduction in glaucoma-related neurodegeneration in experimental conditions by diminishing oxidative stress, lowering ocular hypertension, alleviating retinal ganglion cell loss, and enhancing reparative processes such as myelin development and neuronal regeneration [36]. These findings have substantiated the broader notion that glaucoma management may benefit from supplementary neuroprotective approaches that address oxidative damage and mitochondrial susceptibility, thereby strengthening the rationale for mitochondrial biomodulation strategies that maintain RGC homeostasis in addition to IOP reduction alone [36].

In our retrospective study, approximately 70% of glaucoma patients who received oral nicotinamide riboside (NR) and berberine supplementation for 6 months showed stabilization or a slight improvement in mean deviation (MD) of the visual field. In addition, a subset of these patients demonstrated a slight but measurable increase in retinal nerve fiber layer (RNFL) thickness. These observations suggest a potential direct neuroprotective effect on retinal ganglion cells (RGCs) and their axons. In progressive glaucoma, one would expect a continuous and progressive deterioration of MD and a thinning of RNFL secondary to continued damage and loss of RGC. Therefore, the halting of MD’s decline and hints of improvement are encouraging. The slight RNFL thickening observed, which remains below optimal values, may indicate reduced axonal stress and perhaps the recovery of damaged but not completely dead neurons.

A short-term increase in RNFL (retinal nerve fiber layer) thickness should not be mistaken for actual axonal regeneration—especially within a six-month window, which is biologically improbable in cases of established glaucomatous optic nerve damage. More realistically, these fluctuations likely stem from technical variables, such as inherent OCT measurement noise, inconsistencies in layer segmentation, slight differences in scan alignment, or even shifts in signal strength between visits. That said, subtle physiological changes may also play a role. For instance, reduced metabolic stress or improved mitochondrial function might help stabilize at-risk axons and support glial balance, thereby reducing the progression of microstructural changes without truly regenerating lost fibers. In this context, variations in RNFL thickness are better viewed as indicators of structural preservation or stabilization, rather than as evidence of actual tissue regrowth.

Overall, our results are consistent with the hypothesis that targeting mitochondrial health and well-being, as well as cellular metabolism, can modulate the course of glaucomatous disease by enhancing RGC resistance and viability [37].

It should be emphasized that the molecular concepts discussed in this study have not been experimentally validated within our cohort. Accordingly, no direct measurements of NAD^+^ flux, sirtuin activity, AMPK phosphorylation status, mitochondrial bioenergetic function, or NRF2-mediated transcriptional activation were made. However, the interpretation at the level of signaling pathways is presented as a conceptual framework based on models derived from prior experimental evidence in the literature.

The results are consistent with emerging evidence from both animal and human studies on the role of nicotinamide (vitamin B3) in metabolic support in glaucoma. In a landmark study in mice, Williams et al. showed that age-related decline in NAD^+^ levels in RGCs contributes significantly to vulnerability and that high-dose nicotinamide supplementation protects against the development/progression of glaucoma [38]. In this study, 93% of the eyes did not develop glaucoma when high-dose supplementation was combined. This implies that NAD^+^ replenishment represents a powerful neuroprotective strategy [38]. Subsequent studies have confirmed that nicotinamide can fortify RGCs by supporting mitochondrial function, thereby increasing energy production [37]. For example, chronic administration of nicotinamide in a rat model of ocular hypertension successfully buffered the effects on metabolic stress, maintained adequate NAD levels, and preserved both structural integrity of the optic nerve and RGCs without altering intraocular pressure [39]. These promising pre-clinical results have begun to translate into clinical trials. In fact, a recent randomized phase 2 trial in patients with glaucoma showed that oral nicotinamide (1–3 g/day) combined with pyruvate led to a short-term improvement in visual function compared with placebo [40].

Similarly, a crossover study by Hui et al. showed that high-dose nicotinamide supplementation improves retinal function in patients with glaucoma, suggesting increased RGC activity and improved well-being. Interestingly, patients with glaucoma exhibit systemic deficiencies in nicotinamide/NAD^+^ levels [39]. The availability of NAD^+^, both through nicotinamide and its analogues such as NR, can enhance RGC energy metabolism and confer measurable functional benefits [41]. Our observation of the ability to stabilize the visual field in most patients is consistent with what is present in the most widespread scientific literature.

Berberine is the second component of the supplementation under study. Berberine likely exerts complementary neuroprotective effects by modulating cellular stress pathways. As mentioned, berberine is a plant-derived alkaloid that can cross the blood–brain barrier. It has different pleiotropic pharmacological activities, including antioxidant, anti-inflammatory, and anti-apoptotic effects [42]. A key action of berberine is its activation of AMP-activated protein kinase (AMPK). AMPK represents a central energy sensor capable of triggering adaptive responses when the cell is in a “low energy” state. Activation of this kinase triggers mitochondrial biogenesis and enhances autophagy (by eliminating damaged organelles), thereby improving overall neuronal energy efficiency. On the other hand, the ability of berberine to upregulate the transcription factor erythroid nuclear factor 2-related factor 2 (Nrf2) should not be underestimated; Nrf2 plays a fundamental role in orchestrating intracellular antioxidant defenses [41].

Recent biochemical studies indicate the neuroprotective potential of this important cell signaling pathway (AMPK-Nrf2). For example, in a rodent spinal cord injury model, berberine preserved neuronal viability by inducing AMPK phosphorylation and subsequently increasing Nrf2/HO-1 expression, thereby reducing lipid peroxidation and maintaining mitochondrial function [25]. Pharmacological inhibition of AMPK blocked the protective effects of berberine in that model. These data indicate that AMPK activation is critical for its neuroprotective effect [25].

Although direct data on the use of berberine in glaucoma patients are still lacking in the literature, these preclinical data indicate a role of primary importance in the ability to strengthen resistance to cellular stress and consequently provide a strong rationale for clinical application in glaucoma patients. In this context, berberine’s ability to activate Nrf2 may help counteract oxidative damage affecting the optic nerve [23]. Similarly, by activating AMPK, berberine can improve the energy homeostasis of RGCs and stimulate protective processes (such as mitophagy and mitochondrial biogenesis). The effects of berberine thus complement those of NAD. Combining supplementation has been proposed as a potentially complementary strategy, whereby NAD^+^ support and regulation of stress-response signaling may enhance mitochondrial resilience. Nonetheless, synergistic interaction has not been evaluated in this retrospective cohort because groups receiving NR alone or berberine alone are unavailable. Consequently, the cumulative effect remains observational and hypothesis-generating, necessitating validation through controlled research to compare individual components and their combinations.

**Figure 3:**
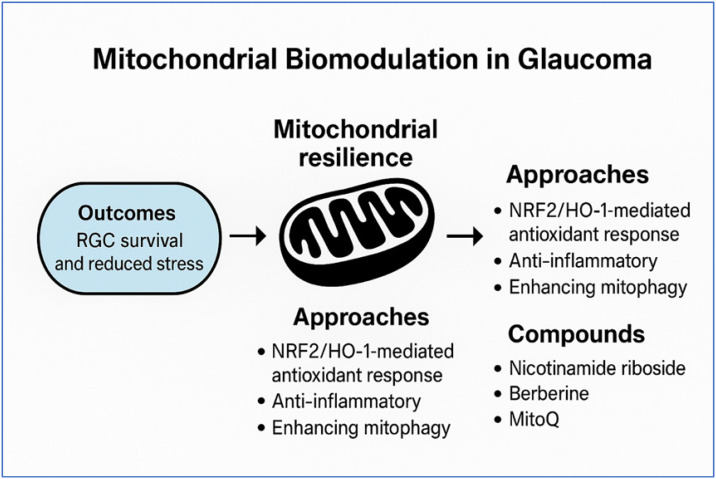
Mitochondrial biomodulation in glaucoma—conceptual framework. Schematic showing therapeutic approaches—NRF2/HO-1-mediated antioxidant response, anti-inflammatory effects, and enhancement of mitophagy—converging on mitochondrial resilience, with expected outcomes of retinal ganglion cell (RGC) survival and reduced oxidative stress. Candidate compounds include nicotinamide riboside, berberine, and MitoQ. Conceptual figure; not based on individual patient data.

The therapeutic rationale for mitochondrial biomodulation is depicted in Figure 3.

## 4. Future Directions

This study paves the way for several hypotheses for future research. It would be useful to conduct prospective randomized controlled trials to more rigorously evaluate the efficacy of nicotinamide/NR and berberine supplementation in patients with glaucoma. Such studies should include control groups (placebo or standard supplementation) and, ideally, be conducted in a double-blind manner to minimize bias. For example, a multicenter study could randomize patients with progressive glaucoma to receive NR and berberine supplementation versus a placebo in addition to hypotonic therapy performed to lower IOP. It would also be useful to conduct a longer follow-up to determine, in depth, whether mitochondrial biomodulation simply delays functional loss or can alter the course of the disease in the long term. Furthermore, future studies should include, in addition to visual field indices and RNFL thickness, electrophysiological measures of RGC function (e.g., ERG pattern) and, perhaps, advanced imaging of the optic nerve to detect subtle neuroprotective effects.

An extended follow-up beyond the trial period, performed by us, could assess whether the effects of treatment persist or whether periodic supplementation is necessary to maintain neuroprotection. Given that glaucoma is a heterogeneous disease, future research could also explore patterns that indicate which subpopulations benefit most from it. It is conceivable that patients with normotension glaucoma (where mitochondrial dysfunction and vascular factors are pronounced) or those with documented systemic oxidative stress may benefit more from this supplementation therapy.

Individual approaches should be studied separately: NR and berberine could be tested individually to determine whether one component exerts most of the effect, or whether the combination of the two molecules is truly synergistic. Other mitochondria-targeting agents (e.g., coenzyme Q10, omega-3 fatty acids) or complementary pathways (e.g., citicoline or neurotrophic factors) could also be combined in a multimodal neuroprotective strategy. In conclusion, our retrospective findings provide a rationale and momentum for new studies of neuroprotection in glaucoma. If confirmed, the support of mitochondrial function and redox balance in RGCs by nutraceuticals such as nicotinamide riboside and berberine could represent a valid adjuvant for reducing IOP and preventing visual field deterioration, directly addressing the neurodegenerative aspect of glaucoma.

## 5. Materials and Methods

**Figure 4:**
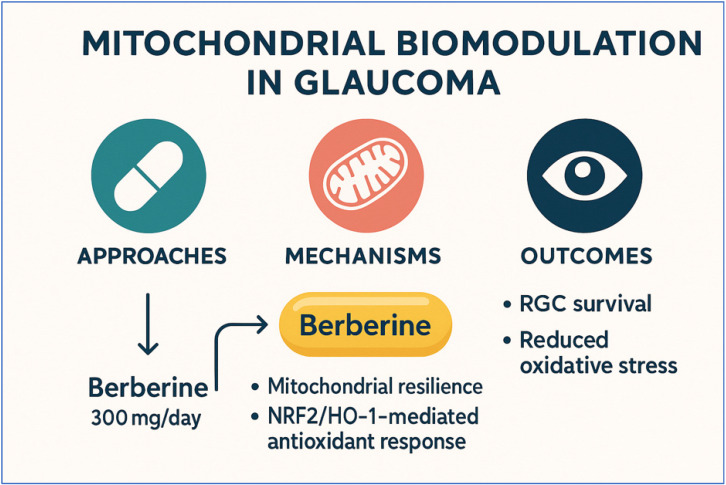
Mitochondrial biomodulation in glaucoma—berberine-focused schematic. Didactic summary highlighting berberine 300 mg/day as a modulator of mitochondrial resilience and the NRF2/HO-1-mediated antioxidant response, within an approaches–mechanisms–outcomes framework. Expected outcomes include retinal ganglion cell (RGC) survival and reduced oxidative stress. Conceptual illustration; not derived from individual patient data.

This observational retrospective study examined the medical records of patients with primary open-angle glaucoma who received, in addition to standard hypotensive therapy, oral supplementation with a standardized nutraceutical formulation. The supplement used contained nicotinamide riboside (300 mg), Berberis aristata extract (300 mg), and niacin (54 mg). This supplementation was administered once daily for six months. The therapeutic rationale for mitochondrial biomodulation is outlined in Figure 4.

We conducted a retrospective, interventional, non-randomized study at the Eye Center “G.B. Morgagni-DSV” (Catania, Italy) from January 2024 to August 2025. All procedures complied with the Declaration of Helsinki and Good Clinical Practice. Visual field testing and OCT examinations were considered essential components of care. The oral supplement is not considered a drug, can be purchased without a prescription, and has been available in Europe since 2023. The Institutional Review Board and Ethics Committee of the Eye Center “G.B. Morgagni-DSV” was informed, which confirmed that approval was not needed for treating patients with routine and approved standard care, for the use of data collected from this study to prepare this study, and to publish the results. Written informed consent was obtained from all participants. All patients provided written informed consent to the use of their anonymized data for research purposes and scientific publications. A total of 41 patients with a diagnosis of primary open-angle glaucoma were retrospectively analyzed. The study cohort included 16 males (39.0%) and 25 females (61.0%), with a mean age of 61.5 ± 14.0 years (range: 21–83 years). All patients were on stable topical antihypertensive therapy for at least 12 months prior to inclusion and exhibited no acute ocular inflammation or systemic conditions that could interfere with mitochondrial metabolism.

### 5.1. Study Population

Eligible patients were adults diagnosed with glaucoma based on morphological changes in the optic nerve, visual field defects, and a history of elevated intraocular pressure (IOP). Only patients who performed a minimum follow-up of six months were included. Visual field examinations and RNFL OCT examination were performed at baseline and post-treatment. Baseline was defined as the most recent clinical evaluation conducted immediately before commencing oral supplementation. At baseline, patients had maintained a consistent topical hypotensive regimen, which had not been altered over the six-month monitoring period unless clinically necessary. Consequently, subsequent evaluations indicate the results obtained with supplementation as an adjunct to continuous standard pharmaceutical treatment. Patients with coexisting eye diseases (e.g., diabetic retinopathy or age-related macular degeneration), those who underwent intraocular surgery during the observation period (excluding uncomplicated cataract surgery), and those with systemic conditions that could confound the results were excluded.

### 5.2. Clinical Assessments

Structural and functional outcomes were analyzed at baseline and at the six-month follow-up. Optical coherence tomography (OCT): The study of the thickness of the peripapillary retinal nerve fiber layer (RNFL) was carried out using spectral domain OCT equipment (Zeiss Cirrus HD-OCT 6000, Carl Zeiss Meditec, Dublin, CA, USA). The automated image segmentation was reviewed by an experienced ophthalmologist who ensured the accuracy of the examination. Visual Field Testing: Visual field examination was performed using the standard SITA Humphrey Field Analyzer (HFA) 24-2 protocol. The mean deviation (MD) value was recorded as the primary visual field parameter. Of the tests conducted, only those that met the reliability criteria (fixation losses < 20%, false positives < 15%, false negatives < 15%) were considered valid and included.

### 5.3. Data Analysis

The primary endpoint was stabilization or improvement in visual field MD at six months compared to the baseline. Secondary endpoints included changes in RNFL thickness over the same period. Given the retrospective design and the limited sample size, no formal hypothesis testing was performed. The results are presented as observational trends rather than inferential statistics.

### 5.4. Limitations

First, visual field indices, such as MD, exhibit intrinsic test–retest variability that increases with defect severity and is suboptimal in reliability. In stable glaucomatous eyes, long-term fluctuations in MD are typically within approximately 0.3–1.3 dB at the 99% confidence level, depending on the stage of damage, and short-term variability is generally below 1–1.5 dB [43]. In large glaucoma cohorts, analyses of paired visual fields taken within short intervals have shown a root-mean-square deviation of approximately 4 ± 2.5 dB [28]. Second, only a limited number of fields were available per eye, and we did not include a run-in or learning phase; residual learning effects or regression to the mean may therefore have contributed to part of the observed improvement. In addition, RNFL measurements obtained by OCT are also subject to test–retest variability. Test–retest variability must be considered when interpreting structural or functional changes. For example, Wadhwani M et al. reported a 95% limits-of-agreement of approximately −3.6 to +4.1 µm for average RNFL thickness using Cirrus HD-OCT in healthy eyes [44]. Therefore, the observed mean increase in RNFL in our cohort should be interpreted with caution. Motion artifacts, scan centration, and signal strength variations may further influence repeatability [45,46]. Finally, this was a single-center retrospective series with a small sample size and no randomized control group; therefore, the findings should be interpreted with caution and validated in larger prospective cohorts. Moreover, limitations to the retrospective analysis undermined the possibility of controlling for parallel comparator groups taking individual constituents of the supplement (e.g., nicotinamide riboside alone, berberine alone or niacin alone), and a corresponding placebo control was missing. Therefore, it was not possible to draw causal inferences or attribute observed effects to specific ingredients. The observed clinical associations should therefore be considered as associative findings, requiring verification in well-powered, prospective controlled trials.

## 6. Conclusions

This retrospective analysis provides preliminary evidence that daily oral supplementation with nicotinamide, riboside, and Berberis, for at least 6 months, can prevent visual field deterioration in patients with primary open-angle glaucoma. The stabilization of the mean visual field deviation observed in the majority of treated individuals, together with a slight, albeit nonsignificant, increase in RNFL thickness in a subgroup, suggests a potential effect on the disease that extends beyond the reduction in intraocular pressure.

From a clinical perspective, these results underscore the growing relevance of mitochondrial biomodulation as an adjunctive therapeutic strategy in the management of glaucoma. Conventional therapies used to lower intraocular pressure remain the cornerstone of glaucoma treatment. However, as clinical practice shows, a significant percentage of patients continue to show progression of glaucomatous damage despite the fact that IOP is well-controlled within desirable values. This translates into clinical practice in two ways. First, nutritional support could be considered in patients who present with progressive disease despite the optimal control of IOP, identifying especially those patients who have systemic risk factors for mitochondrial dysfunction or oxidative stress. Second, these findings underscore the importance of a multifactorial approach in glaucoma therapy. The importance of neuroprotection in patients with glaucoma is increasingly evident.

However, as mentioned above, this study has limitations, such as a retrospective design, the absence of a control group, and a relatively short follow-up period. Prospective randomized trials involving a larger cohort of patients are therefore needed to confirm efficacy, precisely define the optimal dose, and, if possible, identify the patient subgroups most likely to benefit from treatment.

In conclusion, although preliminary, our results add to the evidence already present in the literature, indicating the importance of targeted therapies that present mitochondria as therapeutic targets. These adjuvant treatments have now proven to be fundamental in the treatment of glaucomatous disease. If future controlled studies confirm these effects, supplementation with nicotinamide riboside and Berberis aristata could represent a safe, affordable, and clinically valuable addition to the therapeutic armamentarium for glaucomatous optic neuropathy.

## Figures and Tables

**Table 1:** Changes in RNFL thickness and mean deviation (MD) before and after treatment.

Parameter	Baseline (Mean ± SD)	After Treatment (Mean ± SD)	*p*-Value
RNFL thickness (µm)	54.78 ± 16.32	59.11 ± 16.80	<0.001
MD (dB)	7.20 ± 4.54	4.16 ± 2.79	<0.001

## Data Availability

Data is unavailable due to privacy or ethical restrictions.

## References

[1] Resnikoff S., Pascolini D., Etya’Ale D., Kocur I., Pararajasegaram R., Pokharel G.P., Mariotti S.P. (2004). Global data on visual impairment in the year 2002. Bull. World Health Organ..

[2] Si Z., Fan Y., Wang M., Zhao J., Zhang Y., Liu D., Zheng Y. (2025). The role of RGC degeneration in the pathogenesis of glaucoma. Int. J. Biol. Sci..

[3] Yu D.-Y., Cringle S.J., Balaratnasingam C., Morgan W.H., Yu P.K., Su E.-N. (2013). Retinal ganglion cells: Energetics, compartmentation, axonal transport, cytoskeletons and vulnerability. Prog. Retin. Eye Res..

[4] Anderson D.R., Drance S.M., Schulzer M. (2003). Factors that predict the benefit of lowering intraocular pressure in normal tension glaucoma. Am. J. Ophthalmol..

[5] Gupta N., Ang L.-C., de Tilly L.N., Bidaisee L., Yücel Y.H. (2006). Human glaucoma and neural degeneration in intracranial optic nerve, lateral geniculate nucleus, and visual cortex. Br. J. Ophthalmol..

[6] Muench N.A., Patel S., Maes M.E., Donahue R.J., Ikeda A., Nickells R.W. (2021). The Influence of Mitochondrial Dynamics and Function on Retinal Ganglion Cell Susceptibility in Optic Nerve Disease. Cells.

[7] Weinreb R.N., Aung T., Medeiros F.A. (2014). The Pathophysiology and Treatment of Glaucoma: A review. JAMA.

[8] Guedes-Dias P., Holzbaur E.L.F. (2019). Axonal transport: Driving synaptic function. Science.

[9] Wilkison S.J., Bright C.L., Vancini R., Song D.J., Bomze H.M., Cartoni R. (2021). Local Accumulation of Axonal Mitochondria in the Optic Nerve Glial Lamina Precedes Myelination. Front. Neuroanat..

[10] Killer H., Pircher A. (2018). Normal tension glaucoma: Review of current understanding and mechanisms of the pathogenesis. Eye.

[11] WoldeMussie E., Ruiz G., Wijono M., A Wheeler L. (2001). Neuroprotection of retinal ganglion cells by brimonidine in rats with laser-induced chronic ocular hypertension. Investig. Ophthalmol. Vis. Sci..

[12] Marangoz D., Guzel E., Eyuboglu S., Gumusel A., Seckin I., Ciftci F., Yilmaz B., Yalvac I. (2017). Comparison of the neuroprotective effects of brimonidine tartrate and melatonin on retinal ganglion cells. Int. Ophthalmol..

[13] Risner M.L., Pasini S., McGrady N.R., D’aLessandro K.B., Yao V., Cooper M.L., Calkins D.J. (2021). Neuroprotection by Wld(S) depends on retinal ganglion cell type and age in glaucoma. Mol. Neurodegener..

[14] Xie N., Zhang L., Gao W., Huang C., Huber P.E., Zhou X., Li C., Shen G., Zou B. (2020). NAD^+^ metabolism: Pathophysiologic mechanisms and therapeutic potential. Signal Transduct. Target. Ther..

[15] D’angelo A., Vitiello L., Lixi F., Abbinante G., Coppola A., Gagliardi V., Pellegrino A., Giannaccare G. (2024). Optic Nerve Neuroprotection in Glaucoma: A Narrative Review. J. Clin. Med..

[16] Williams A., Ramsden D. (2005). Nicotinamide: A double edged sword. Park. Relat. Disord..

[17] Nzoughet J.K., de la Barca J.M.C., Guehlouz K., Leruez S., Coulbault L., Allouche S., Bocca C., Muller J., Amati-Bonneau P., Gohier P. (2019). Nicotinamide Deficiency in Primary Open-Angle Glaucoma. Investig. Ophthalmol. Vis. Sci..

[18] Hui F., Tang J., Williams P.A., McGuinness M.B., Hadoux X., Casson R.J., Coote M., Trounce I.A., Martin K.R., van Wijngaarden P. (2020). Improvement in inner retinal function in glaucoma with nicotinamide (vitamin B3) supplementation: A crossover randomized clinical trial. Clin. Exp. Ophthalmol..

[19] Bohnen N.I., Yarnall A.J., Weil R.S., Moro E., Moehle M.S., Borghammer P., Bedard M.-A., Albin R.L. (2022). Cholinergic system changes in Parkinson’s disease: Emerging therapeutic approaches. Lancet Neurol..

[20] Hampel H., Mesulam M.-M., Cuello A.C., Khachaturian A.S., Vergallo A., Farlow M.R., Snyder P.J., Giacobini E., Khachaturian Z.S. (2019). Revisiting the Cholinergic Hypothesis in Alzheimer’s Disease: Emerging Evidence from Translational and Clinical Research. J. Prev. Alzheimer’s Dis..

[21] Cheng Z., Kang C., Che S., Su J., Sun Q., Ge T., Guo Y., Lv J., Sun Z., Yang W. (2022). Berberine: A Promising Treatment for Neurodegenerative Diseases. Front. Pharmacol..

[22] Li Z., Geng Y.-N., Jiang J.-D., Kong W.-J. (2014). Antioxidant and Anti-Inflammatory Activities of Berberine in the Treatment of Diabetes Mellitus. Evid.-Based Complement. Altern. Med..

[23] Song D., Hao J., Fan D. (2020). Biological properties and clinical applications of berberine. Front. Med..

[24] Pires E.N.S., Frozza R.L., Hoppe J.B., Menezes B.d.M., Salbego C.G. (2014). Berberine was neuroprotective against an in vitro model of brain ischemia: Survival and apoptosis pathways involved. Brain Res..

[25] Tian E., Sharma G., Dai C. (2023). Neuroprotective Properties of Berberine: Molecular Mechanisms and Clinical Implications. Antioxidants.

[26] Imenshahidi M., Hosseinzadeh H. (2019). Berberine and barberry (*Berberis vulgaris*): A clinical review. Phytother. Res..

[27] Khoshandam A., Imenshahidi M., Hosseinzadeh H. (2022). Pharmacokinetic of berberine, the main constituent of *Berberis vulgaris* L.: A comprehensive review. Phytother. Res..

[28] Ahmed T., Gilani A.-U., Abdollahi M., Daglia M., Nabavi S.F., Nabavi S.M. (2015). Berberine and neurodegeneration: A review of literature. Pharmacol. Rep..

[29] Fan J., Zhang K., Jin Y., Li B., Gao S., Zhu J., Cui R. (2019). Pharmacological effects of berberine on mood disorders. J. Cell. Mol. Med..

[30] Visalli F., Capobianco M., Cappellani F., Rapisarda L., Spinello A., Avitabile A., Cannizzaro L., Gagliano C., Zeppieri M. (2026). Mitochondrial Health Through Nicotinamide Riboside and Berberine: Shared Pathways and Therapeutic Potential. Int. J. Mol. Sci..

[31] Kitaoka Y., Sase K., Tsukahara C., Fujita N., Arizono I., Takagi H. (2020). Axonal Protection by Nicotinamide Riboside via SIRT1-Autophagy Pathway in TNF-Induced Optic Nerve Degeneration. Mol. Neurobiol..

[32] Sun G.-F., Qu X.-H., Jiang L.-P., Chen Z.-P., Wang T., Han X.-J. (2024). The mechanisms of natural products for eye disorders by targeting mitochondrial dysfunction. Front. Pharmacol..

[33] DiNicolantonio J.J., McCarty M.F., Assanga S.I., Lujan L.L., O’KEefe J.H. (2022). Ferulic acid and berberine, via Sirt1 and AMPK, may act as cell cleansing promoters of healthy longevity. Open Heart.

[34] Zong Y., Li H., Liao P., Chen L., Pan Y., Zheng Y., Zhang C., Liu D., Zheng M., Gao J. (2024). Mitochondrial dysfunction: Mechanisms and advances in therapy. Signal Transduct. Target. Ther..

[35] Luo L.-J., Nguyen D.D., Lai J.-Y. (2020). Benzoic acid derivative-modified chitosan-*g*-poly(*N*-isopropylacrylamide): Methoxylation effects and pharmacological treatments of Glaucoma-related neurodegeneration. J. Control. Release.

[36] Li S., Gaur U., Chong C.-M., Lin S., Fang J., Zeng Z., Wang H., Zheng W. (2018). Berberine Protects Human Retinal Pigment Epithelial Cells from Hydrogen Peroxide-Induced Oxidative Damage through Activation of AMPK. Int. J. Mol. Sci..

[37] Leung C.K.S., Ren S.T., Chan P.P.M., Wan K.H.N., Kam A.K.W., Lai G.W.K., Chiu V.S.M., Ko M.W.L., Yiu C.K.F., Yu M.C.Y. (2022). Nicotinamide riboside as a neuroprotective therapy for glaucoma: Study protocol for a randomized, double-blind, placebo-control trial. Trials.

[38] Tribble J.R., Otmani A., Sun S., Ellis S.A., Cimaglia G., Vohra R., Jöe M., Lardner E., Venkataraman A.P., Domínguez-Vicent A. (2021). Nicotinamide provides neuroprotection in glaucoma by protecting against mitochondrial and metabolic dysfunction. Redox Biol..

[39] Williams P.A., Harder J.M., Foxworth N.E., Cochran K.E., Philip V.M., Porciatti V., Smithies O., John S.W.M. (2017). Vitamin B_3_ modulates mitochondrial vulnerability and prevents glaucoma in aged mice. Science.

[40] Cimaglia G., Tribble J.R., Votruba M., Williams P.A., Morgan J.E. (2024). Oral nicotinamide provides robust, dose-dependent structural and metabolic neuroprotection of retinal ganglion cells in experimental glaucoma. Acta Neuropathol. Commun..

[41] De Moraes C.G., John S.W.M., Williams P.A., Blumberg D.M., Cioffi G.A., Liebmann J.M. (2022). Nicotinamide and Pyruvate for Neuroenhancement in Open-Angle Glaucoma. JAMA Ophthalmol..

[42] Harder J.M., Guymer C., Wood J.P.M., Daskalaki E., Chidlow G., Zhang C., Balasubramanian R., Cardozo B.H., Foxworth N.E., Deering K.E. (2020). Disturbed glucose and pyruvate metabolism in glaucoma with neuroprotection by pyruvate or rapamycin. Proc. Natl. Acad. Sci. USA.

[43] Ma H., Xing C., Wei H., Li Y., Wang L., Liu S., Wu Q., Sun C., Ning G. (2024). Berberine attenuates neuronal ferroptosis via the AMPK–NRF2–HO-1-signaling pathway in spinal cord-injured rats. Int. Immunopharmacol..

[44] Tattersall C.L., A Vernon S., Menon G.J. (2007). Mean deviation fluctuation in eyes with stable Humphrey 24-2 visual fields. Eye.

[45] Choi E.Y., Li D., Fan Y., Pasquale L.R., Shen L.Q., Boland M.V., Ramulu P., Yousefi S., De Moraes C.G., Wellik S.R. (2021). Predicting Global Test–Retest Variability of Visual Fields in Glaucoma. Ophthalmol. Glaucoma.

[46] Wadhwani M., Bali S.J., Satyapal R., Angmo D., Sharma R., Pandey V., Dada T. (2015). Test-Retest Variability of Retinal Nerve Fiber Layer Thickness and Macular Ganglion Cell-Inner Plexiform Layer Thickness Measurements Using Spectral-Domain Optical Coherence Tomography. J. Glaucoma.

